# The oscillatory entrainment of virtual pitch perception

**DOI:** 10.3389/fpsyg.2013.00210

**Published:** 2013-04-25

**Authors:** Aleksandar Aksentijevic, Anthony Northeast, Daniel Canty, Mark A. Elliott

**Affiliations:** ^1^Department of Psychology, University of RoehamptonLondon, UK; ^2^Department of Psychology, University of SurreySurrey, UK; ^3^School of Psychology, National University of IrelandGalway, Ireland

**Keywords:** gamma-band, virtual pitch, pitch perception, pitch coding, harmonic templates, oscillatory priming

## Abstract

Evidence suggests that synchronized brain oscillations in the low gamma range (around 33 Hz) are involved in the perceptual integration of harmonic complex tones. This process involves the binding of harmonic components into “harmonic templates” – neural structures responsible for pitch coding in the brain. We investigated the hypothesis that oscillatory harmonic binding promotes a change in pitch perception style from spectral (frequency) to virtual (relational). Using oscillatory priming we asked 24 participants to judge as rapidly as possible, the direction of an ambiguous target with ascending spectral and descending virtual contour. They made significantly more virtual responses when primed at 29, 31, and 33 Hz and when the first target tone was harmonically related to the prime, suggesting that neural synchronization in the low gamma range could facilitate a shift toward virtual pitch processing.

## INTRODUCTION

Processing the pitch of harmonic complex tones represents one of the most important research topics in auditory perception and cognitive neuroscience (e.g., Patterson et al., 2002; Penagos et al., 2004; Micheyl and Oxenham, 2007; McLachlan, 2011). There is accumulating evidence that the perceptual integration of disparate harmonic components in primary auditory cortex (PAC) involves the formation of harmonic templates (Brunstrom and Roberts, 1998; Lin and Hartmann, 1998; Shamma and Klein, 2000; Bendor, 2011), that is, networks of medium-range connections between topographically non-adjacent neural loci which code harmonically related frequencies (e.g., Kadia and Wang, 2003). One issue of particular concern is the mechanism which mediates the transformation of spectral (frequency) information into “virtual” Gestalt-like pitch percepts (Terhardt, 1974, 1989). The “Gestaltness” of a virtual pitch percept is reflected in the fact that it arises only when the constituent components are related to each other in a specific way.

**FIGURE 1:**
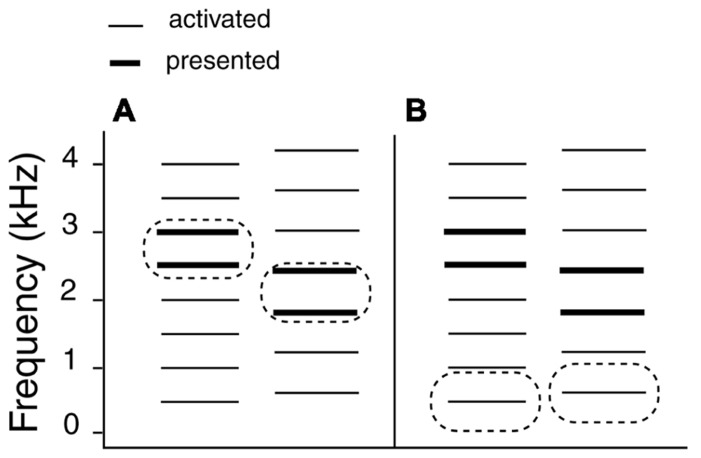
**Two modes of pitch perception**. A pair of pure tones (2500 + 3000 Hz) is succeeded by another (1800 + 2400 Hz). The tone pairs activate harmonic templates of different fundamental frequencies (500 and 600 Hz, respectively). **(A)** Spectral mode. A descending melodic contour is derived from the frequencies of audible components. **(B)** Virtual mode. An ascending contour is derived from the missing fundamental frequencies based on the mutual distance of individual harmonic components.

The fact that a single stimulus can provide two mutually exclusive forms of pitch information (spectral and virtual) offers a convenient way of investigating the dynamics of harmonic pitch integration. Typically, such stimuli consist of pairs of two-frequency tones (see **Figure 1**). The frequencies are selected so that the frequencies in the second tone are lower than those in the first. At the same time, the frequency distance between components increases in the second tone (Smoorenburg, 1970) producing two competing contours – a falling spectral (1A) and a rising virtual (1B) contour. The former is extracted from the frequencies of successive components and the latter is defined by the frequency of the missing fundamental – the inaudible first harmonic of a relevant series. The spectral mode relies on the computation of the frequency differences whereas the virtual mode involves abstraction of tonotopic relationships in the sense that harmonic distance becomes the new unit of information for the purposes of computation and comparison.

Studies using such ambiguous stimuli reveal considerable inter-individual differences in listening style. While some listeners are capable of hearing virtual pitch, others derive pitch information from the spectral contour (the frequency difference between successive harmonics) and this preference appears to be stable over time (Patel and Balaban, 2001). Moreover, the relationship between the two modes of pitch perception is unidirectional: Pantev et al. (2001) found that once trained to hear virtual pitch, listeners have difficulty reverting to the spectral listening mode (see also Deutsch and Steeger, 1978). According to Winkler et al. (1994), virtual pitch is coded in sensory memory and takes longer to process as compared to spectral pitch. While listeners can be described as adopting either a spectral or a virtual mode, repeated exposure to harmonic tones can lead to an irreversible switch from the former to the latter.

A recent investigation of harmonic pitch integration using a priming paradigm (Aksentijevic et al., 2011) suggests that priming in the low gamma range (33 Hz) is involved in the synchronization of templates involved in the coding of target frequencies harmonically related to the carrier frequency of the prime. The priming causes an “inharmonic pop-out,” that is, an increased salience of targets that are not harmonically related to the prime. In a second study, (Aksentijevic and Elliott, under revision) the inharmonic pop-out was observed in participants with no musical training but not in musically trained participants. This suggests that prolonged exposure to harmonic input creates permanent connections between harmonic template components. Aksentijevic and Elliott argue that once these connections have been formed, transient oscillatory synchronization becomes less relevant as a mechanism for binding.

Support for this view comes from a study by Schulte et al. (2002), in which listeners were exposed to an ambiguous melody over a period of several hours. The melody consisted of a sequence of pairs of pure tones which were arranged such that while their spectral contour produced a descending motif, the virtual melody was that of the well-known folk tune “Frère Jacques.” After several hours of listening to the stimulus, all participants were able to recognize Frère Jacques. This shift to a virtual mode was accompanied by a functional reorganization of the PAC – by the anatomical redistribution of the evoked auditory gamma-band response (aGBR; e.g., Galambos, 1992). Following exposure, the aGBR ostensibly migrated away from the core toward the belt area in secondary auditory cortex suggesting that the shift to a virtual mode represents the consolidation of an oscillatory “harmonic memory” in the PAC (Jutras and Buffalo, 2010; Headley and Weinberger, 2011).

In the current study we employed ambiguous targets to investigate the hypothesis that rate-specific priming in the low gamma range (around 33 Hz) would facilitate a perceptual shift toward the virtual listening mode. Spectral down – virtual up targets (SDVU) consisted of a pair of harmonics of the 500 Hz harmonic series (2500 + 3000 Hz) followed by a pair of harmonics of the 600 Hz series (1800 + 2400 Hz; see **Figure 1**). An SUVD target represented the reversed arrangement of the SDVU target. We conjectured a shift toward the virtual mode would be one consequence of the binding of components of a relevant harmonic template by means of oscillatory priming, which would promote the salience of the relational (virtual) pitch information relative to that carried by the spectral contour. In order to test this, we initially sought to replicate the original rate-specific priming effect found by Aksentijevic et al. (2011) at or around 33 Hz.

In the original study, the binding effect was demonstrated indirectly – via the increased salience of *inharmonic *targets. Here, the two versions of the target stimulus permitted a direct test of the binding hypothesis. In the SUVD condition, the first target tone (2500 + 3000 Hz) is harmonically related to the prime. If the hypothesis that oscillatory priming affects only harmonic loci is correct, the oscillatory binding should facilitate virtual responses to SUVD targets which inherit the harmonic information entrained by the prime.

## MATERIALS AND METHODS

### PARTICIPANTS

The study was approved by the Ethics Committee of the Department of Psychology, University of Roehampton, and conformed to the Helsinki Declaration on Human Rights. Twenty four undergraduate volunteers (five male) took part in the experiment (mean age 21.71 years, standard deviation 6.7 years, with one participant substantially older than the remaining participants). Two participants were left-handed and one was ambidextrous. Seven participants had received from 2 to 14 years of musical training. All participants reported normal hearing and none possessed absolute pitch. Apart from one drummer, all musically trained participants received training on pitched instruments including voice. All participants provided informed consent and received credits for taking part in the study.

### APPARATUS AND STIMULI

Experimental trial and stimulus generation, as well as response timing were controlled by an IBM compatible computer with sound signals generated by an internal stereo sound card. Trial presentation and data collection were implemented using Superlab (v.4, Cedrus). The stimuli were presented diotically via Sennheiser HD 265 linear headphones.

**FIGURE 2:**
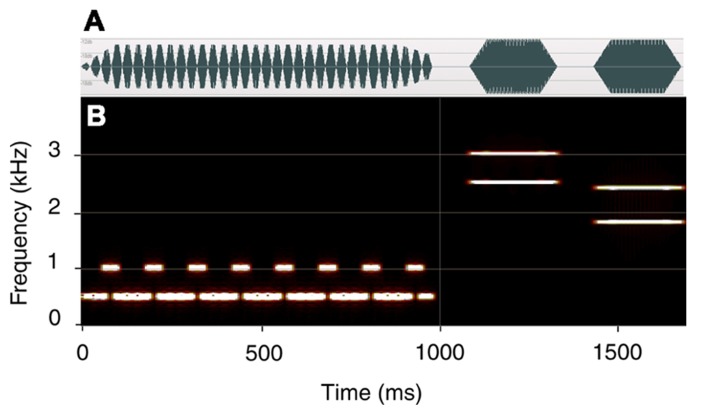
**Experimental trial**. The prime is presented at the rate of 33 pps and the ISI is 100 ms. A spectral down – virtual up (SDVU) target is shown. **(A)** the waveform; **(B)** time-frequency plot. The first target tone carries the fifth and sixth harmonic of 500 Hz (2500 + 3000 Hz) and is harmonically related to the prime. The second target tone carries the third and fourth harmonics of 600 Hz (1800 + 2400 Hz). A reversed target (SUVD) was presented on 50% of the trials.

Each trial consisted of two parts (see **Figure 2**). In the first, a 1000 ms tone-pip train (prime) was presented, designed so as to generate a gamma-band oscillatory steady state response (SSR; Galambos et al., 1981; Galambos and Makeig, 1992; Thut et al., 2011). Presentation rate was defined by the duration of individual tone pips. The prime was presented at 29, 31, 33, 35, or 37 pips-per-second (pps). The individual pips were created using NCH tone generator (Version 3.00; NCH Software) and were onset- and offset-ramped with ramps that were symmetrical and had a plateau of 33% of the overall period (**Figure 1A**). The trains were created using WavePad sound editor (NCH Software).

The prime consisted of a repeating four-pip sequence, with every first pip carrying 1000 Hz, and the remaining three pips carrying 500 Hz. As demonstrated by Aksentijevic et al. (2011), this structure was critical in generating an internal oscillatory model responsible for the observed effects. Specifically, the regular reset of the oscillatory representation of the 500 Hz template by a 1000 Hz pip led to a periodic oscillatory model whose temporal properties could be examined very precisely. At 33 pps, the supra-threshold amplitude of the internal oscillatory model (expected to affect participants’ responses) was predicted and confirmed at inter-stimulus intervals (ISIs) of 106 and 256 ms (± 5 ms). Pip duration was a reciprocal of presentation rate (e.g., 30.3 ms at 33 pps). Following an ISI of either 100 or 400 ms, a target was presented consisting of two 250 ms tones separated by an ISI of 100 ms. In the original study, the priming effect was observed at ISIs of 100 and 250 ms. While we estimated the persistence of the oscillatory representation at about 400 ms, we failed to observe any effects at ISIs above approximately 250 ms. Consequently, the two ISIs were chosen a) in order to replicate the original finding of a rate-specific effect at 100 ms ISI and b) confirm the absence of the effect at longer intervals. Each target complex contained two frequencies and was symmetrically ramped (rise and fall time 50 ms). The first tone carried 2500 and 3000 Hz (the fifth and sixth harmonics of the baseline prime frequency of 500 Hz) while the second carried 1800 and 2400 Hz (the third and fourth harmonics of 600 Hz). This produces two perceptual solutions. If listeners derive contour from the spectral information (frequency of successive harmonics), they hear a descending contour. Conversely, if they compute contour from the harmonic relationship between concurrent harmonics (virtual pitch), they hear an ascending contour. This target is labeled SDVU. It should also be noted that the first tone of the SDVU target was harmonically, and the second inharmonically, related to the prime. A reversed version of the target (spectral up – virtual down; SUVD) was presented on 50% of the trials in order to control for the harmonic relationship between the prime and target and increase stimulus variability. To recapitulate, the two target conditions were generated by inverting a single two-tone stimulus. Both versions of the target were presented an equal number of times.

### DESIGN

The study employed a within-subjects design with factors ISI (100 ms, 400 ms), rate (29, 31, 33, 35, 37 pps) and target direction (SDVU, SUVD). There were 10 trials per condition resulting in 200 trials per participant. Trials were presented pseudo-randomly for each participant across five blocks of 40 trials per block. Each session commenced with a 40-trial practice block.

### PROCEDURE

The experiment was conducted in a sound-attenuated experimental room. Stimulus intensity was held constant throughout [average 60 dB sound pressure level (SPL); A weighting; average error 2 dB SPL], examined using an artificial ear and an Adastra analog sound level meter, (Model 952.422, slow response), with the shape of the stimuli examined using WavePad sound editor. Each trial commenced with a 500 ms cross appearing at the center of the screen. Participants were instructed to focus on the target and indicate as quickly as possible if the target was going up or down by pressing the appropriate keyboard key with a finger of their dominant hand. In conjunction with target configuration, this task would produce response profiles corresponding to individual propensity to hear virtual pitch. Response time was measured from target onset and a new trial was initiated by the response. An individual session lasted approximately 15 min.

## RESULTS

**FIGURE 3:**
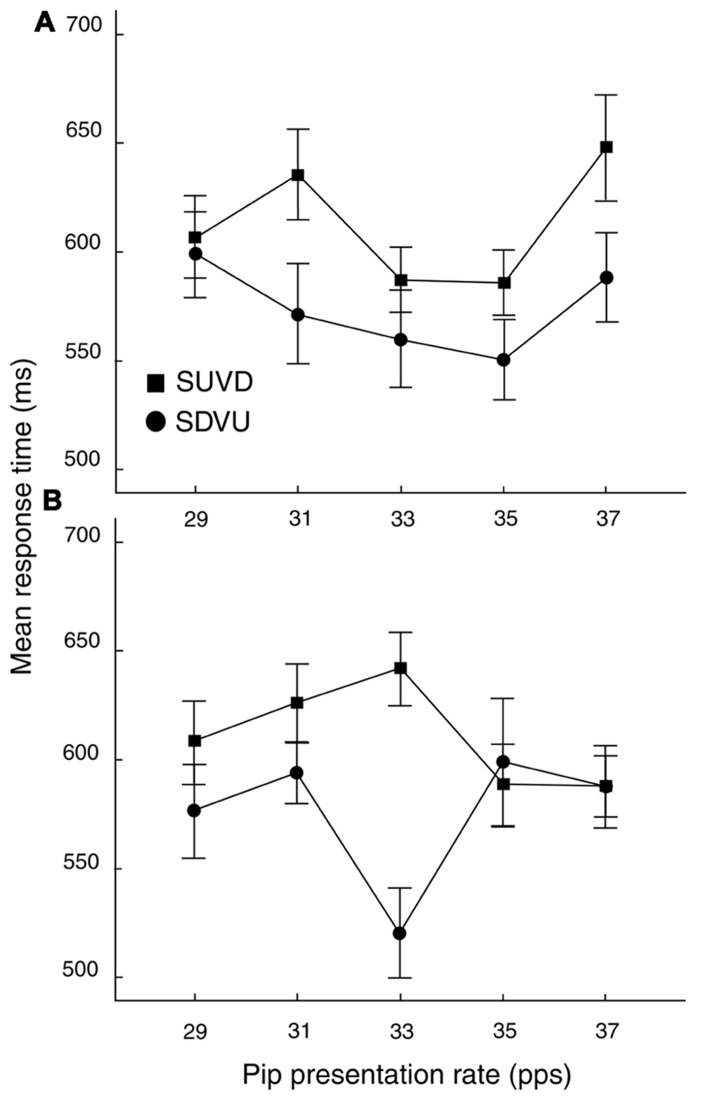
**Mean RTs (± 1 SEM) as a function of ISI, rate and target**. **(A)** Rate by target interaction at 100 ms ISI. **(B)** Rate by target type interaction at 400 ms ISI. SDVU = spectral down – virtual up; SUVD = spectral up – virtual down. SEM values were computed using the procedure described by Loftus and Masson (1994).

Prior to analysis, 43 trials (out of 4800) were removed because they contained task-irrelevant responses. Data were analyzed in two stages. First, an omnibus analysis was performed on response-time (RT) data in order to test against the results of Aksentijevic et al. (2011) and to establish the presence and frequency of a rate-specific priming effect in the current paradigm. For this, anti-logs of the means of the log-transformed RT distributions were subject to a three-way repeated-measures ANOVA with factors ISI (100, 400 ms), rate (29, 31, 33, 35, and 37 pps) and target direction (SDVU, SUVD). The main effect of target direction was significant [*F*(1,23) = 10.12, *MSE *= 14432.1, *p* < .01; $\eta_{p}^{2}$ =.31] with SDVU targets being registered more rapidly (mean difference = 39 ms, *SEM* = 13 ms). The three-way interaction was also significant [*F*(4,92) = 3.03, *MSE* = 8011.66, *p* < .05; $\eta_{p}^{2}$ =.12 (see **Figure 3**)], resulting from a significant difference in the effect of rate at the two ISIs as confirmed by the inspection of Bonferroni-adjusted *post-hoc* comparisons. Specifically, at 100 ms ISI, there was a significant RT advantage for SDVU targets at 31 pps (difference: 64 ms, *SEM* = 24.47, *p* < .05), while by contrast at 400 ms ISI, there was a large and significant 118 ms (*SEM* = 25.82, *p* < .001) RT advantage for SDVU targets that followed a 33 pps prime consistent with the rate-specific inharmonic pop-out reported by Aksentijevic et al. (2011).

Our main hypothesis concerned the idea that gamma-band entrainment at specific rates would promote the shift toward virtual perceptual mode. In order to test this, participants’ responses were coded with regard to their listening style. “Down” responses to SUVD targets and “Up” responses to SDVU targets were coded as “virtual”. The opposite, reciprocal combination was coded as “spectral”. Overall, the percentage of virtual responses was 49.7% (2362 out of 4757 responses), suggesting an equal representation of the two listening styles. Three participants made close to 100% virtual responses while three made less than 5% virtual responses. Four participants made an approximately equal number of spectral and virtual responses. This is consistent with the observation of a considerable inter-subject variability with respect to listening mode (e.g., Deutsch and Steeger, 1978; Patel and Balaban, 2001; Schneider et al., 2005) and suggests that the current task could be used as a convenient diagnostic test of listening style. Some musically trained participants produced more spectral responses suggesting that musical training does not automatically lead to a virtual shift. This is in broad agreement with the finding by Schneider et al. (2005) of a bimodal distribution of listening style with the majority of listeners (mostly musicians), exhibiting a relatively strong preference for either style and some showing a mixed profile.

**FIGURE 4:**
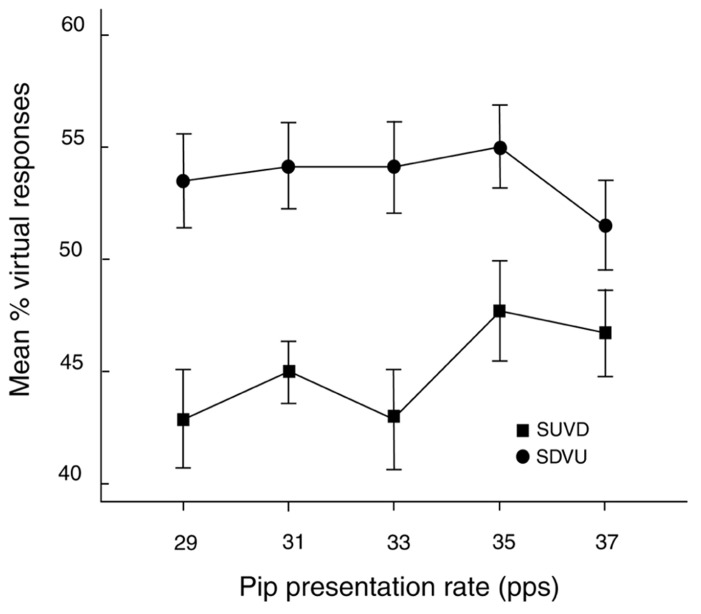
**Mean percentage (± 1 SEM) of virtual responses as a function of rate and target**. SDVU = spectral down – virtual up; SUVD = spectral up – virtual down.

A three-way ANOVA with factors ISI (100, 400 ms), rate (29, 31, 33, 35, 37 pps) and target direction (SDVU, SUVD) was performed on the percentages of virtual responses across all conditions (**Figure 4**). The main effect of target direction was significant [*F*(1,23) = 5.16, *MSE* = .137, *p* < .05; $\eta_{p}^{2}$ =.18] indicating that more virtual responses were made to SDVU targets (mean difference = 6.8%, *SEM* = 3.3%). In addition, this effect was influenced by presentation rate as indicated by a significant rate by target direction interaction [*F*(4,92) = 3.57, *MSE* = .009, *p* < .01; $\eta_{p}^{2}$ =.13]. Inspection with Bonferroni correction revealed this to be caused by a greater proportion of virtual responses to SDVU relative to SUVD targets at 29 (11%, *SEM* = .04%, *p* < .01), 31 (7%, *SEM* = .03%, *p* < .05) and 33 pps (11%, *SEM* = .04%, *p* < .05) but not at higher rates.

To examine the relationship between RT performance and listening style, the RT ANOVA was rerun with listening style as a between-subject factor. “Listening style” was defined in terms of preponderance of virtual or spectral responses. The four listeners who had made between 50 and 55% virtual or spectral responses were removed from analysis and the others were assigned to either a “virtual” or “spectral” condition. Apart from the obvious main effect of listening style [*F*(1,18) = 93.55, *MSE* = 20.52, p < .001; $\eta_{p}^{2}$ =.84], no significant effects of the latter factor were observed suggesting that the relationship between the two tasks was complex and would require further investigation. First, the interaction between listening style and RT facilitation might be observable only in “intermediate” listeners – those who are most likely to be affected by priming. We might not have had enough of these responders to observe the relationship between RTs and listening style and this justifies further experimental investigation. Second, the RT effects of priming might be at least partly independent of the virtual shift. To illustrate, there is evidence that virtual pitch perception requires more time relative to spectral pitch perception (e.g., Winkler et al., 1994). So, the RT effects could be confounded by the slowing down with respect to the virtual responses. It is worth noting that the same result was obtained with a stricter (75%) exclusion criterion.

## DISCUSSION

The current study investigated the effects of oscillatory gamma-band priming (Aksentijevic et al., 2011) on the perceptual classification of ambiguous melodies. As shown in previous research (Schulte et al., 2002), a spectral perceptual mode is characterized by a focus on the frequency components that constitute complex tones. Conversely, virtual listening relies on the Gestalt properties of the stimulus, namely the relationships between harmonics. In most normal listening situations, this dichotomy is not relevant because complex tones (e.g., musical instrument tones or voice) are present in their entirety. In situations in which the two modes are brought into conflict, listeners need to make a decision based on the relative strength of either representation. In the present study, a two-tone stimulus was presented that could be interpreted either spectrally or virtually. Virtual responses were defined in reference to a specific target. This meant that response probabilities could not be established a priori (as would have been the case with a up/down responses to a binary independent variable) but depended on the listening style – individual propensity to hear spectral or virtual pitch. This meant that our results reflected the composition of the sample, that is, the presence of participants whose responses could be affected by priming. A sample comprising a large number of strong virtual or spectral listeners would likely not have produced significant effects.

The principal finding was that more virtual responses were made to SDVU relative to SUVD targets when the primes were presented at 29, 31, and 33 pps and this effect was independent of the interval between the prime and the target. The results suggest that the priming in the low gamma range could promote the binding of harmonically related components into a template thereby increasing the perceptual salience of the virtual Gestalt. The observed dependence on the type of target suggests that the presence of the first, harmonically related target tone played a role in the shift by re-establishing the entrained harmonic representation.

**FIGURE 5:**
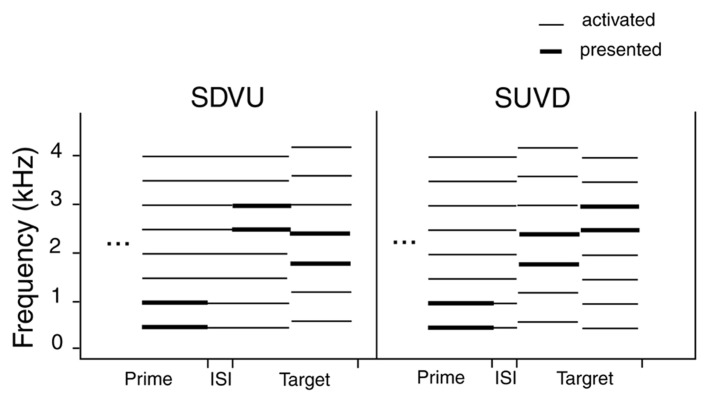
**Relationship between harmonic templates activated by the stimuli as a function of target type (SDVU = spectral down – virtual up; SUVD = spectral up – virtual down)**. Thick lines denote presented frequencies and the thin ones the frequencies that were activated by the stimuli.

**Figure 5** illustrates the relationship between harmonic templates activated by the stimuli. The left-hand side illustrates the SDVU condition. The prime (500 + 1000 Hz) activates the 500 Hz template, which in turn results in an oscillatory representation that persists following the prime offset (top left). The first target tone (2500 + 3000 Hz) fits into the active template helping to maintain the representation. The second tone (1800 + 2400 Hz) does not fit into the active template causing a reset in the oscillatory representation. This increases the salience of the conflicting 600 Hz template and facilitates the virtual “up” decision, but only at 29, 31, and 33 pps in broad agreement with Aksentijevic et al. (2011). The reinforcement of the oscillatory 500 Hz template representation by the first target tone contributes to the virtual shift. By contrast, the first tone in a SUVD target (1800 + 2400 Hz) would have disrupted the 500 Hz template entrained by the prime immediately leading to its re-establishment following the second tone. Since the response was based on comparing the second target tone to the first one, the time was not sufficient for the latter to establish an alternative harmonic representation, resulting in ambiguity. This in turn would have reduced the salience of the second target tone relative to the SDVU condition.

The absence of ISI effects in direction judgment data could be explained in terms of the difference between RT and direction aspects of the task. As shown by Aksentijevic et al. (2011), rate-specific RT effects were not mediated by consciousness (participants were unable to report the harmonicity relationship of the target to the prime). This is not the case with up-down decisions which require conscious control. While our results suggest that the processing advantage conferred by gamma priming benefits decisional processes, it is unlikely that this benefit derives directly from sensitivity to the temporal code of the entrainer. Instead, this code raises the value of a structural criterion in the stimulus that influences decisional processes. It is likely that decisions are affected by an increased salience of the virtual pitch representation, influenced in turn by the temporal parameters of the entrained oscillatory representation whose effects are ISI specific.

The importance of priming rate was corroborated by the time-specific RT enhancement mediated by target and ISI. The results indicate that SDVU targets were processed faster while producing more virtual responses when primed in the low gamma range. At the shorter ISI of 100 ms, responding was faster for SDVU targets if they were primed at 31 pps. At the longer ISI, the inharmonic advantage was observed at 33 pps. Given the temporal specificity of the stimuli, this is in agreement with previous findings of an inharmonic RT advantage for stimuli presented at 33 pps (Aksentijevic et al., 2011). Aksentijevic et al. (2011) reported reliable gamma-band priming for ISIs of up to 250 ms. The current unexpected finding of a rate-specific inharmonic facilitation at 400 ms ISI indicates that residual oscillatory activity in the low gamma range persists for longer than previously found and that it can be resynchronized by non-modulated targets. While in the original study, both the prime and the target consisted of tone-pips, here targets contained non-modulated sinusoids. Given the extreme temporal specificity of the effect (± 2 ms), a possible explanation is that the presence of the uninterrupted target sinusoid facilitates the resynchronization of the oscillatory representation. Nevertheless, further research is needed to elucidate the relationship between this late effect and the temporal parameters of the entrained oscillatory model.

The current findings of a rate-related shift in listening mode are consistent with an interaction between two (as of now putative) systems, namely, the pitch extraction system located in the PAC (lateral Heschl’s gyrus; Schneider et al., 2005; Bendor and Wang, 2008, 2010) and the contour processing system located in the secondary auditory areas (planum temporale and planum polare; Puschmann et al., 2010). While contour can be extracted from spectral and virtual pitch information, it is hypothesized that the consolidation of harmonic templates underpinned by gamma oscillations leads to the change in the contour processing system such that it switches to computing the contour information from the distances between individual harmonics (the pitch of the fundamental) rather than their position on the tonotopic axis.

## CONCLUSION

The current study provides the first piece of behavioral evidence that synchronized brain oscillations in the low-gamma range could promote the shift toward the virtual listening mode. Our results might have important implications for pitch perception research in two ways. First, the current task appears to represent a simple and useful diagnostic of pitch processing style. Subject to validation, this could be applied in a wide variety of experimental and applied settings in which perception plays an important role (e.g., auditory and musical development, autism, musicianship, hemispheric lateralization). Second, our results suggest that changes in pitch processing can be effected rapidly, without prolonged exposure and/or training. A systematic multi-method investigation of the time course, persistence and reversibility of the observed priming effect would afford important insights into the temporal and spatial parameters of pitch processing.

At the same time, the current design in which virtual responses were reciprocals of spectral responses could not exclude the possibility that gamma priming somehow inhibited spectral responses. While this is not likely, further studies are needed to elucidate this issue. Future research will also seek to corroborate this evidence by combining behavioral and electroencephalography (EEG) data. It will also examine the role of the relationship between spectral and virtual frequencies in modulating the intensity and time course of this virtual shift.

## Conflict of Interest Statement

The authors declare that the research was conducted in the absence of any commercial or financial relationships that could be construed as a potential conflict of interest.
